# Involvement of Technical Reasoning More Than Functional Knowledge in Development of Tool Use in Childhood

**DOI:** 10.3389/fpsyg.2016.01625

**Published:** 2016-11-08

**Authors:** Chrystelle Remigereau, Arnaud Roy, Orianne Costini, François Osiurak, Christophe Jarry, Didier Le Gall

**Affiliations:** ^1^Department of Psychology, Psychology Laboratory, LUNAM, University of AngersAngers, France; ^2^Reference Center for Learning Disabilities, Nantes University HospitalNantes, France; ^3^Neurofibromatosis Clinic, Nantes University HospitalNantes, France; ^4^CNRS UMR 8158, Psychology Laboratory of PerceptionParis, France; ^5^Neurology Service, Rothschild Ophthalmological FoundationParis, France; ^6^Laboratory for the Study of Cognitive Mechanisms, University of LyonLyon, France; ^7^University Institute of FranceParis, France; ^8^Department of Neurology, Angers University HospitalAngers, France

**Keywords:** tool use, child development, mechanical problem-solving, functional knowledge, school-aged children

## Abstract

It is well-known that even toddlers are able to manipulate tools in an appropriate manner according to their physical properties. The ability of children to make novel tools in order to solve problems is, however, surprisingly limited. In adults, mechanical problem solving (MPS) has been proposed to be supported by “technical reasoning skills,” which are thought to be involved in every situation requiring the use of a tool (whether conventional or unusual). The aim of this study was to investigate the typical development of real tool use (RTU) skills and its link with technical reasoning abilities in healthy children. Three experimental tasks were adapted from those used with adults: MPS (three different apparatus), RTU (10 familiar tool-object pairs), and functional knowledge (FK; 10 functional picture matching with familiar tools previously used). The tasks were administered to 85 healthy children divided into six age groups (from 6 to 14 years of age). The results revealed that RTU (*p* = 0.01) and MPS skills improve with age, even if this improvement differs according to the apparatus for the latter (*p* < 0.01 for the Hook task and *p* < 0.05 for the Sloping task). Results also showed that MPS is a better predictor of RTU than FK, with a significant and greater weight (importance weight: 0.65; Estimate ± Standard Error: 0.27 ± 0.08). Ours findings suggest that RTU and technical reasoning develop jointly in children, independently from development of FK. In addition, technical reasoning appears partially operative from the age of six onward, even though the outcome of these skills depends of the context in which they are applied (i.e., the type of apparatus).

## Introduction

Compared to other animal species, tool use is very common in human cultures and one of the specific features of this behavior is that it evolves in a cumulative manner (Tennie et al., 2009; Jarry et al., 2016). Innovations are transmitted from one individual to another and from one generation to the next through social learning (Beck et al., 2012; Osiurak et al., 2016). In this regard, from 2 years of age onward, children start to adequately use tools that they encounter in their environment (i.e., conventional tool use; Lockman, 2000; Vaesen, 2012; Cutting et al., 2014), and this occurs in particular through their abilities to imitate (i.e., adaptation to social uses). Interestingly, the ability to solve mechanical problems by using novel tools (e.g., an unknown apparatus such as the Hook task, see below) may take place later, at about 8 years of age (Beck et al., 2011). These findings suggest that real tool use (RTU; e.g., the daily use of a spoon by a toddler to eat) and mechanical problem solving (MPS) skills could be based on cognitive mechanisms of a different nature. This is partly inconsistent with the technical reasoning hypothesis mainly developed from the study of left brain-damaged patients, according to which familiar tool use and MPS should be supported by common cognitive mechanisms (Le Gall, 1998; Osiurak et al., 2010; Osiurak, 2014; Osiurak and Badets, 2016). The main goal of the present study is to address this issue, by exploring how children aged from 6 to 14 years perform on RTU and MPS tasks.

### Development of Tool-Making Abilities in Childhood

Lockman (2000) proposed that, from a developmental perspective, the emergence of tool use before 1 year of age develops in the absence of optimal representational thinking (i.e., symbolic). Several authors have hence assumed that human tool use is derived from perceptual-motor behavior implemented by children at a very young age: exploration of surfaces/textures/sounds, trial and error strategies, affordance perception, grip selection, etc. (van Leeuwen et al., 1994; Kahrs and Lockman, 2014). Recent studies have, however, reported a striking dissociation between these early skills and the subsequent emergence of tool-making abilities in children. Studies have shown on multiple occasions that making a functional tool independently (i.e., without any demonstration, called “tool innovation” below) is nearly impossible in healthy children before the age of 5 years (Beck et al., 2011, 2014; Cutting et al., 2011) irrespective of their socio-cultural environment (Nielsen et al., 2014). The task commonly employed consists in recovering a target that is lodged inside a transparent tube and that cannot be reached directly by hand (e.g., the Hook task, Unbending task). The making of a new functional tool, by modification of available items (for example a hook from a straight metal rod), does not occur spontaneously until late in child development: only half of 8-year-old children can do so (Beck et al., 2011). Furthermore, this ability is subject to an age effect up to 16–17 years of age. While children aged 4 years are not able to generate a new solution themselves, they are nonetheless readily able to select a suitable pre-made tool (for instance a hooked rod versus a straight rod), thus indicating adequate analysis of the possible use for such tools. In addition, a prior exploration phase of the tool’s physical properties (e.g., softness, stiffness and bendability) does not lead to an improvement in the ability to innovate (Beck et al., 2011; Gardiner et al., 2012). On the other hand, following a demonstration by the investigator of how to make a functional tool (i.e., demonstrated how to bend a rod into a hook), the children can generally reproduce and use tools from age 3 onward, thereby revealing that they have sufficient dexterity to solve the problem (Chappell et al., 2013). Thus, the dexterity is sufficient from age 3, and the selection of a functional tool seems to occur in children from age 4, for solving a problem involving tool making (Beck et al., 2014). So, it is not likely that these factors explain a development of tool innovation abilities up to 16–17 years of age. In addition, among the children who can achieve the task, it appears that tool innovation is not the result of learning by trial and error, since they are able to make it first-off or after a single unfruitful attempt (Cutting et al., 2011).

### Underlying Processes of Tool Innovation in Children

The previously presented results also revealed different developmental timetables according to the experimental conditions that were considered: tool manufacturing (i.e., making a functional tool following a demonstration, from age 3 onward) versus tool innovation (i.e., spontaneously making a functional tool, from age 5 onward), suggesting involvement of distinct cognitive processes. Limited consideration has been given, however, to the “innovative” nature of the proposed apparatus due to the single solution expected by the investigator: alteration of the available tool with which the children act on the unknown entities (i.e., the Hook task can only be solved if the child manufactures a hook). Gardiner et al. (2012) also reported better abilities among young children (3 years of age) *to select* a pre-made functional tool than *to use it* to solve a mechanical problem (e.g., extraction of a small stuffed animal from a transparent box). Unlike the work of Beck and collaborators, in this study no prior transformation of the material was necessary to solve the problem. This dissociation is interpreted as a failure to adequately relate the selected tool with the apparatus, while affordances of the tool should be adequately apparent, thereby allowing its selection (Gardiner et al., 2012).

Thus, the question of specificity of the underlying cognitive processes engaged in these tool innovation tests remains unanswered. Does the alteration of the material to innovate a new tool (as in the Hook task) imply the same cognitive abilities as those engaged in relationships between a pre-made tool with an apparatus (like in Gardiner’s study)? To answer this question, it would be relevant to compare the developmental curves characterizing the performances achieved with familiar tool use as well as with various types of mechanical apparatus (for which some require alteration of the material) by the same sample of children.

### Contributions of Studies in Adults

Neuropsychological studies in adults can also shed light on the understanding of tool innovation in children, notably because similar experimental devices are commonly employed to assess tool use skills in patients with apraxia. Specifically, several studies have shown in left brain-damaged patients a strong association between conventional tool use (in a prototypical or unusual manner) and MPS (Goldenberg and Hagmann, 1998; Osiurak et al., 2009; for review see Osiurak and Badets, 2016; Reynaud et al., 2016). This has led to the formulation of the hypothesis that technical reasoning is involved in any tool use, whether conventional or new/unusual (Le Gall, 1998; Osiurak et al., 2010; Osiurak, 2014). This cognitive process refers to the capacity to adapt the purpose of an apparatus (e.g., to punch holes) and the underlying technical process (e.g., one item less dense than the other, such as paper sheet/staple). The means and ends are abstract and relative principles, because they do not correspond with the physical reality. Rather they are reconstructed *de novo* according to each form of use. The same material (e.g., wood) can provide distinct technical properties (e.g., resilience, opaqueness, flammability) according to the intended purpose. The same technical property (e.g., resilience) can also be presented by different materials (e.g., glass, metal, plastic). In this context, Jarry et al. (2013, p. 2323) assumed that the inability to use tools could reflect difficulties regarding differentiating and relating relevant technical means (e.g., dense, permeable, resilient, etc.), for a given outcome (e.g., cutting, etching, etc.). For example, “the lead of a pencil is brittle when applied to paper, but not to leather.”

It has also been established that knowledge of prototypical tool use (semantic memory) is involved in tool use, particularly for adaptation to social uses (e.g., to use scissors “like everyone else”; Vaesen, 2012; see also Defeyter and German, 2003 for the concept of “functional fixedness” in children). Yet, while this functional knowledge (FK) might be useful for conventional tools use (i.e., for which prior use has occurred), it is not necessary for intrinsic use and does not suffice to account for everyday uses, which generally require uncommon procedures and the use of non-familiar tools (see also Vaesen, 2012). Baumard et al. (2014) pointed out that apraxic patients exhibiting impairments of technical reasoning (e.g., on a sequential MPS test) systematically have pronounced difficulties using tools in a conventional way. Although this conclusion was drawn from adults, it stresses that the cognitive processes involved in solving mechanical problems, also participate in effective use of tools in other contexts.

### Overview of the Present Study

The aim of our study was to explore the developmental link between RTU and MPS skills in healthy children. As suggested, children could be able to use tools in a conventional way before developing considerable MPS skills. This is partly inconsistent with the neuropsychological literature, which mentions a strong link between these two aspects. Three different apparatus were used for the assessment of MPS skills, because the success rates in children for these tasks can differ according to the transparency mechanical relationships and the required degree of tool manipulation (Gardiner et al., 2012). So, at a theoretical level, it appears fundamental to understand how the different forms of tool use skills evolve with age. From a clinical perspective, this is also a fundamental question, since impairment of tool use is frequently described in various etiological contexts with children, such as developmental coordination disorders, cerebral palsy, premature birth, autism (O’Hare et al., 1999; Wunsch et al., 2013; Geldof et al., 2016). Yet the cognitive processes that underlie these impairments are rarely analyzed, due to a lack of available assessment tests in particular. Besides, children were also assessed on a task assessing FK, notably because it has been suggested that this knowledge could be the support for familiar tool use (Vaesen, 2012). Our study focused on children aged from 6 to 14 years, a period in which significant age effects on tool innovation have been noted in previous research, with the emergence of alternative problem-solving strategies from the age of 8 years old (Beck et al., 2011). To our knowledge, no study has investigated normal childhood developmental curves up to the age of 14 for these tests.

## Materials and Methods

### Participants

Following formal approval of the study protocol by the School Inspection Services, a volunteer cohort of 85 children aged 6 up to and including 14 years of age was recruited between January 2014 and June 2015 from the French school system (from first grade to third grade). Once permission was obtained from the school principal and the teachers, all of the children in the class were given the option to participate in the study, provided that the children did not exhibit any of the exclusion criteria: sensory impairments (visual or auditory), their proficiency with the French language was insufficient to allow them to perform the tests, neurodevelopmental or psychiatric pathologies, suspected or proven learning disorders, premature birth (prior to 37 weeks of gestation), or lack of parental consent. The total sample (mean age of 10 years and 3 months, SD: 2 years and 5 months) was divided into six experimental groups according to their ages: 6–7 year olds, 8 year olds, 9 year olds, 10 year olds, 11–12 year olds, and 13–14 year olds.

### Materials

The assessment of effective tool use (conventional or new) was performed using tests developed for a population of adults with cerebral lesions (Jarry et al., 2013; Lesourd et al., 2016), adapted in the present study for use with children.

#### Real Tool Use (RTU)

**FIGURE 1:**
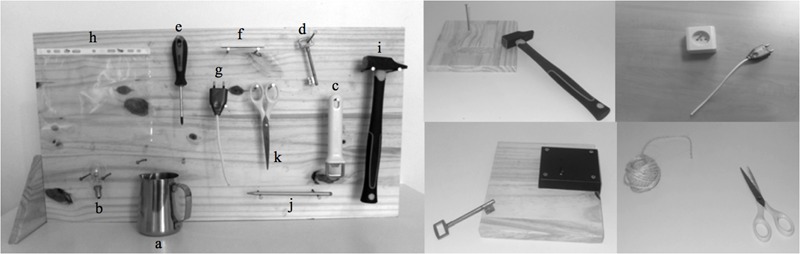
**The real tool use test.** The left picture represents the vertical “tool panel” used for the presentation of the tools (a, jug; b, light bulb; c, bottle cap opener; d, key; e, screwdriver; f, piece of chalk; g, electrical plug; h, plastic sleeve; i, hammer; j, lead pencil; k, scissors). The right picture show examples of tools and the corresponding objects upon which they can be used (i.e., glass, desk lamp, capped glass bottle, lock on a wooden board, screw in a wooden board, slate, electrical socket, ringbinder, nail in a wooden board, pencil sharpener, coil of hemp yarn, respectively).

For this tool use test, the apparatus was comprised of 11 familiar tools displayed simultaneously on a vertical board (see **Figure 1**). The corresponding objects with which the tools could be used were arranged one by one on a table between the child and the vertical board.

Each child was asked to select the tool corresponding with the object presented, and to perform the usual action carried out with the displayed tools and objects. The pair jug/glass was used as a practice item (i.e., assistance with the selection and performance of the action, if necessary) during which the examiner stressed to the children that they needed to fully perform the actions. Performance was video-taped, and one point was awarded if the child exhibited correct use of the object with the appropriate tool within the time limit (i.e., 1 min for each item). No point was awarded if the action was not successfully carried out, according to efficiency criteria defined beforehand (e.g., the position of the dead bolt changed due to the action of the key in the lock, the pencil was sharpened by the pencil sharpener and wood shavings were generated). The maximum possible score was 10 points.

#### Mechanical Problem-Solving (MPS)

**FIGURE 2:**
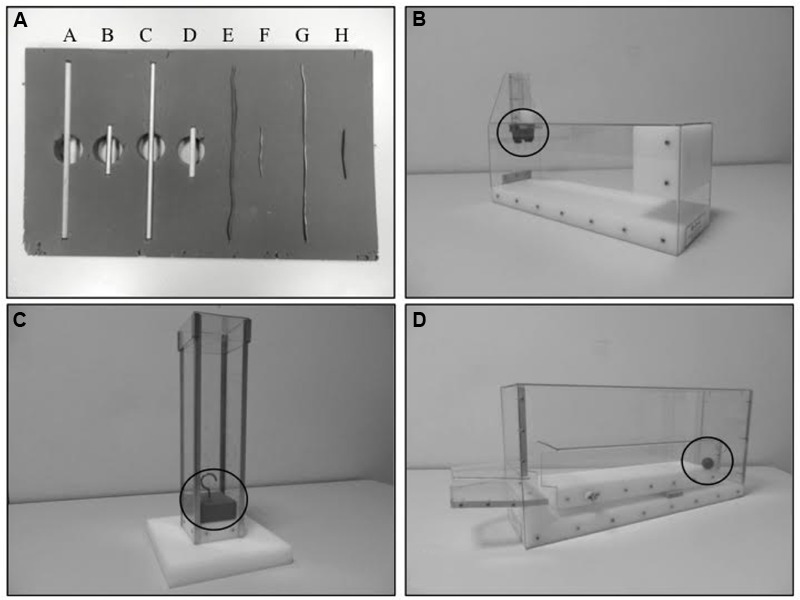
**Apparatus and rods used in the mechanical problem solving tasks.** The black circles denote the wooden targets that the children had to extract from the boxes (derived from Lesourd et al., 2016). **(A)** The displayed rods; **(B)** the Chimney task; **(C)** the Hook task; **(D)** the Sloping task.

For this test, we used three different transparent plastic apparatus, as Lesourd et al. (2016), albeit with multiple choices. The aim of this test was to extract a little red wooden cube or bead (i.e., the targets), which were lodged inside each of the boxes. For each apparatus, the Chimney task (**Figure 2B**), the Hook task (**Figure 2C**), and the Sloping task (**Figure 2D**) required different actions in order to solve the problem (for more details see Lesourd et al., 2016). To do so, the children were provided with the following materials (**Figure 2A**): four 25 cm rods (one was made of wood and the others were either aluminum, copper, or tin) and four 7 cm rods (made of the same physical material). The rods were displayed simultaneously in a row on a table to the right of the subjects. The rods hence had various physical properties, e.g., half were long (i.e., rods A, C, E, and G, as shown in **Figure 2A**) and half were short (i.e., rods B, D, F, and H), half were rigid (i.e., rods A, B, C, and D) and half were flexible (i.e., rods E, F, G, and H).

The participants could perform this test using one or both hands, but the problem could not be solved by directly inserting a hand into the box, by shaking or other physical handling, by trial and error, or by choosing the rods at random. A sequence comprised of two actions was necessary to solve the problem. Children could use several rods to extract the target (i.e., combining several rods or alternating different rods according to the target’s movement in the box), but only two rods were relevant and necessary to solve each problem (see Lesourd et al., 2016 for more details). Before the test, a sample test was performed (i.e., assistance with performing the action, if necessary) so as to ensure that the children had identified the target that needed to be extracted and had adequately understood the aim of the test. The test item consisted of a red wooden bead that needed to be extracted from a rectangular and horizontal tube by pushing it with a wooden rod. The only instruction that was given with the example was: “using the items at your disposal, show me how to recover the target that is lodged in this box. You may turn the box but do not lift it up.” No feedback was given but children were given neutral prompts if required: “keep going, there is a solution.” Performance was video-taped, and the time limit was 3 min for each item. Two variables were collected for this test: a qualitative score in terms of success/failure (“Beck’s rating”) and a quantitatively accurate score based on a 4-point scale (see **Table 1**), which was quite similar to the one used by Jarry et al. (2013). The maximum score was 3 points for each apparatus.

**Table 1:** Accuracy-based scoring system used for mechanical problem solving tasks.

	3 points	2 points	1 point	0 point
The Chimney task		The target had fallen down but was not extracted from the box	The child touched the target with a rod but without causing it to fall down	
*The Hook task	The target was extracted from the box within the time limit, independently of the rod that was used	The target was fully taken off the base but it was not extracted from the box	The child touched the target with a rod but the target was never completely taken off the base	The children touched the box but never the target
The Sloping task		The target was wedged near the opening due to the action of a rod but it was not extracted from the box	The child touched the outer part of the bottom of the seesaw with a rod introduced into the round opening but they did not activate the seesaw	

#### Functional Knowledge (FK)

Four images with different single familiar tools were displayed below the picture of a tool target (target stimuli were the same as those used in the RTU test). The children were asked to choose one of the four response options as the best match for the target, by pointing to it. The test was preceded by one practice trial (with a jug) for which feedback regarding the correctness was given. The target and the correct response were functionally equivalent (e.g., electrical plug and candle). A score of 1 point was awarded if the correct response was given within 20 s, equating with a total possible score of 10 points.

### Procedure

When possible, children were tested at their school (in a quiet room), or at home (in an isolated room, without the parents being present). The tests were systematically administered in a specific ordered manner for each participant: RTU test first, then MPS tasks and finally FK test. This work was included in a broader study regarding gestural development and tool use in children. An anamnestic questionnaire completed by the parents allowed for confirmation prior to the assessment that the criteria for inclusion/exclusion in the study were adhered to. An informed consent form (authorizing the video recordings) was signed by a legal representative for each child, specifying their freedom to withdraw from the study without any prejudice. The rationale and design of the study were detailed in an information pamphlet. The research was conducted in accordance with the University guidelines and the ethical standards established by the Helsinki Declaration.

### Data Analysis

Preliminary statistical analyses were conducted with variance analysis (ANOVAs, Bonferroni *post hoc*) and the Chi-Square test was used to examine the equivalence of the age groups in terms of gender, the parents’ level of education, and laterality. The inspection of MPS, RTU, and FK score distributions revealed that none of them met the conditions for application of parametric tests: non-normal distribution of raw scores (Kolmogorov–Smirnov one-sample test) and non-homogeneity of variances (Levene’s test). Consequently, Kruskal–Wallis ANOVAs were used to examine the effect of the main factor, namely age (six attributes: 6–7 years of age, 8 years of age, 9 years of age, 10 years of age, 11–12 years of age, 13–14 years of age), on MPS performances (three apparatus raw scores), RTU and FK results. *Post hoc* contrasts were computed with Mann–Whitney tests. Friedman ANOVAs were used to examine the effect of the apparatus (different types of boxes) according to age, with Wilcoxon signed-rank tests used for within-group comparisons according to age. Spearman rank correlations were used to study relationships between Age, RTU, MPS, and FK on the whole group (*n* = 85). However, to better examine the influence of MPS and FK on RTU, we used a generalized linear mixed model (GLMM; Burnham et al., 2011), which included MPS and FK as fixed factors, and Age as arandom factor. This analysis was conducted on the whole group (*n* = 85). For this analysis, RTU scores were converted into binomial scores (10: Success; <10: Failure). Akaike’s Information Criterion value corrected for small sample size (AICc) was calculated for the GLMM. Models were ranked in relation to each other using ΔAICc values [Δi = AICc(i) – AICc(min)]. Akaike weights were computed (ωi) to assess the likelihood of the model relative to the other models considered. All models were averaged to calculate predictor estimates and standard errors using full-model averaging method (Burnham et al., 2011). The GLMM analyses and the model-averaged coefficients were respectively computed using the lme4 and MuMIn packages in R 3.1.3. For all analyses, the alpha threshold was fixed at *p* < 0.05. The effect sizes were calculated with *Cohen’s d*, considering the effect as being large from 0.8 and as being medium from 0.5 (Cohen, 1988).

## Results

### Sample Characteristics

Sociodemographic characteristics of the sample are presented in **Table 2**. The age groups were comparable in terms of laterality, according to the Edinburgh Handedness Inventory (Oldfield, 1971; with values between +100 for extreme right hand preference and -100), *F*(5,79) = 0.502, *NS*, and sex ratios, χ^2^(6) = 6.191, *NS*, although there was a significant difference in terms of the parental level of education, *F*(5,79) = 3.766, *p* < 0.01. Bonferroni’s *post hoc* test showed that the parental education level was significantly higher for the 8 year old children than for the 10 and 11–12 years old children. However, no significant correlation was found between the parental education level and scores for the MPS (all *r* < -0.10, *NS*), RTU (*r* = 0.03, *NS*), or the FK (*r* = -0.10, *NS*) test. We hence did not take this variable into account for the statistical analyses.

**Table 2:** General data related to the different age groups of the sample.

	6–7 years (*n* = 17)	8 years (*n* = 12)	9 years (*n* = 10)	10 years (*n* = 15)	11–12 years (*n* = 16)	13–14 years (*n* = 15)
Age (months)	84.94 (6.92)	101.67 (2.49)	110.80 (3.66)	126.07 (3.60)	145.31 (8.22)	167.13 (4.56)
Girls/boys (n)	8/9	8/4	8/2	10/5	8/8	7/8
Edinburgh (quotient of laterality)	81.18 (41.00)	65.00 (54.70)	71.50 (28.46)	82.67 (46.69)	60.63 (53.67)	64.67 (60.54)
PEL (in years)	15.21 (2.88)	16.00 (3.87)	14.15 (2.75)^a^	12.43 (2.04)^a^	12.34 (2.30)^a^	13.10 (3.02)

*All scores correspond to mean scores (with standard deviations in parentheses). PEL, Parental Education Level (mean for both parents). ^a^For one child the PEL was calculated based on the level of education for only one of their parents.*

Similarly, gender had no effect on the children’s performances on the MPS, RTU, and FK tests (Mann–Whitney tests, data not shown). Thus, data from boys and girls were combined for further analyses.

### Age Effect

**FIGURE 3:**
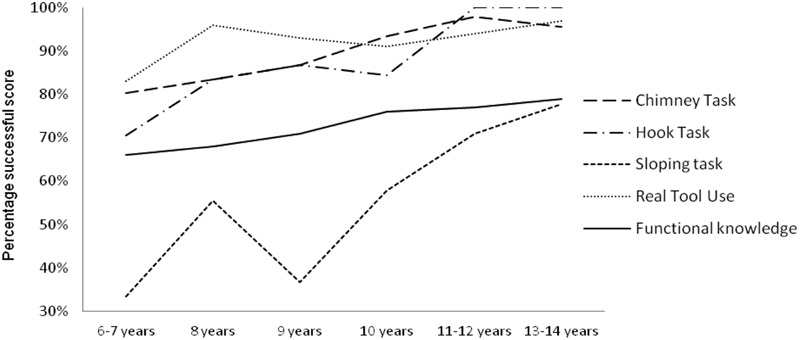
**Developmental course of MPS, RTU, and FK tests (*n* = 85).** Percentage successful score accuracy-based scoring system for the three MPS tasks.

An effect of age was observed on the RTU test, *H*(5,85) = 14.618, *p* = 0.01. *Post hoc* tests were computed and showed that only the youngest children performed worse than children aged 8, 11–12, and 13–14 years of age, with large effect sizes (*Cohen’s d* between 0.87 and 1.30). Furthermore, the successful scores increased with age for the FK, although only a tendency toward significance (*p* = 0.054) was found between the age groups. These results are illustrated by the developmental curves shown in **Figure 3**.

**FIGURE 4:**
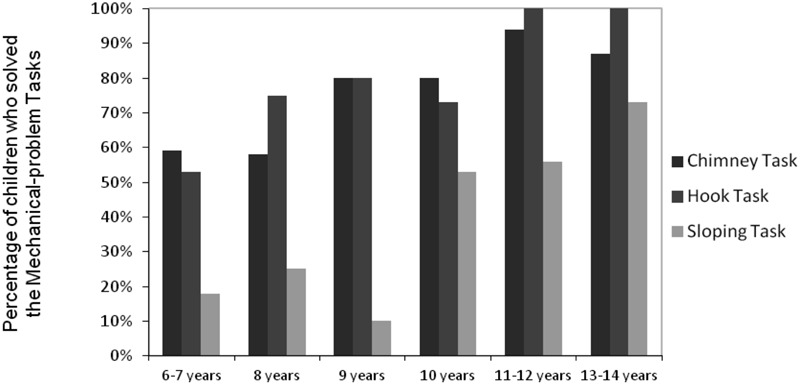
**Dichotomous scores of children according to age for the MPS tasks (Beck’s rating)**.

We compared the qualitative performances (“Beck’s rating”) for the various age groups with the three apparatus for the MPS task using Chi-Square tests. There was a trend for the performance on the Chimney task to improve with age (only 59% of children succeeded at 6–7 years of age, while 94% succeeded at 11–12 years of age), although only two significant differences emerged (see **Figure 4**): more of the 11–12 year-old children succeed than the 6–7 and the 8 year olds (χ^2^ = 5.470, *p* = 0.02; χ^2^ = 5.110, *p* = 0.02, respectively). A greater progression was found with the Hook task: the 11–12 and the 13–14 year-old children were better than the younger 6–7, 8, and 10 year-old (all *ps* < 0.03). Likewise, on the Sloping task, the 10–14 year olds were better than the 6–7 and 9 year olds (all *ps* < 0.03). The number of children who succeeded at this task was also significantly higher among the 13–14 year-old relative to the group of 8 year olds (χ^2^ = 6.240, *p* = 0.01).

Results for the accuracy-based scoring system in the MPS tasks (4-point scale) are presented in **Table 3**. Kruskall-Wallis ANOVAs revealed a main effect of age on two of the three apparatus for the MPS test: the Hook task, *H*(5,85) = 15.683, *p* < 0.01, and the Sloping task, *H*(5,85) = 14.583, *p* < 0.05. There was no effect of age on the quantitative scores for the Chimney task, *H*(5,85) = 9.066, *NS*. For the Hook task, the *post hoc* analyses showed that the maximal successful performance was reached from 11 years of age onward, which is significantly distinct from the performances of the 6–7, 8, and 10-year-old, with large effect sizes (*Cohen’s d* between 0.79 and 1.16). For the Sloping task, comparisons between age groups indicate that the 6–7 and the 9 year olds children performed worse than children aged 11–12 and 13–14 (*Cohen’s d* between 1.05 and 1.22). Additionally, the youngest children (6–7 year-old) performed worse than children aged 8 years on this apparatus, with a medium effect size (*d* = 0.70).

**Table 3:** Mechanical problem solving, real tool use, and functional knowledge accuracy-based scores system according to the age group.

	Chimney task	Hook task	Sloping task	Real tool use	Functional knowledge
6–7 years(G1, *n* = 17)	2.4 (0.9)	2.1 (1.1)	1.0 (1.1)	8.3 (1.4)	6.6 (1.2)
8 years(G2, *n* = 12)	2.5 (0.7)	2.5 (0.9)	1.7 (0.9)	9.6 (0.7)	6.8 (1.7)
9 years(G3, *n* = 10)	2.6 (1.0)	2.6 (0.8)	1.1 (0.7)	9.3 (0.9)	7.1 (1.1)
10 years(G4, *n* = 15)	2.8 (0.4)	2.5 (0.9)	1.7 (1.4)	9.1 (0.8)	7.6 (1.4)
11–12 years(G5, *n* = 16)	2.9 (0.3)	3.0 (0.0)	2.1 (1.0)	9.4 (1.1)	7.7 (1.3)
13–14 years(G6, *n* = 15)	2.9 (0.4)	3.0 (0.0)	2.3 (1.2)	9.7 (0.6)	7.9 (1.1)
*Main effect(H, Kruskal–Wallis)*	9.066	15.683^∗∗^	14.583^∗^	14.618^∗^	10.882
*Groups comparison (U, Mann–Whitney) with Effect size (Cohen’s d)*	–	G1 < G5^∗∗^, G6^∗∗^ (*d* = 1.16) G2 < G5^∗^, G6^∗^ (*d* = 0.79) G4 < G5^∗^, G6^∗^ (*d* = 0.79)	G1 < G2^∗^, G5^∗∗^, G6^∗∗^ (*d* = 0.70) (*d* = 1.05) (*d* = 1.13) G3 < G5^∗^, G6^∗^ (*d* = 1.16) (*d* = 1.22)	G1 < G2^∗∗^, G5^∗^, G6^∗∗^(*d* = 1.17) (*d* = 0.87) (*d* = 1.30)	–

*All scores correspond to mean scores (with standard deviations in parentheses). G1, Group 1; G2, Group 2; G3, Group 3; G4, Group 4; G5, Group 5; G6, Group 6. ^∗^*p* < 0.05; ^∗∗^*p* < 0.01; ^∗∗∗^*p* < 0.001. The effect sizes were calculated with Cohen’s *d*, considering the effect as large from 0.8 and as medium from 0.5*

### Relationships between RTU, MPS, and FK

The correlations between scores for the MPS, FK, and RTU tests are presented in **Table 4**.

**Table 4:** Correlations according to whole sample (*n* = 85) between real tool use, mechanical problem-solving and functional knowledge.

	Age	Real tool use	Mechanical problem solving
Real tool use	0.30^∗∗^		
Mechanical problem solving	0.52^∗∗∗^	0.21^∗^	
Functional knowledge	0.39^∗∗∗^	0.05	0.14

*Spearman correlation coefficients. ^∗^*p* < 0.05; ^∗∗^*p* < 0.01; ^∗∗∗^*p* < 0.001.*

When the different age groups were pooled together (i.e., *n* = 85), three significant correlations were seen between age and RTU/MPS/FK (all *ps* < 0.01). In addition, a significant correlation was also seen between RTU and MPS (*r* = 0.21; *p* = 0.049). Nonetheless, no significant correlation was found between scores for RTU and FK, or between MPS and FK.

As mentioned, the influence of the predictors MPS and FK on RTU was tested using a generalized linear mixed model (GLMM) with Age as a random factor. There were three top candidate models with Δi < 2 (see **Table 5**), the best one being the MPS only model. We also computed model-averaged estimators based on AICc. The only statistically significant predictor of RTU is MPS (Importance weight: 0.65; Estimate ± Standard Error: 0.27 ± 0.08). This variable was significant at the 95% confidence level (the interval did not contain the 0 value).

**Table 5:** Influence of MPS and FK on RTU (GLMM analysis).

Models	*k*	AICc	*Δ_i_*	ω*_i_*
MPS	3	118.67	0.00	0.47
*Intercept*	*2*	*119.86*	*1.19*	*0.26*
*MPS* + *FK*	*4*	*120.64*	*1.97*	*0.18*
FK	3	121.93	3.27	0.09

*k, number of parameters in the model; AICc, Akaike’s information criterion corrected; Δ*_i_*, AIC_c(i)_ – AIC_c(min)_; ω*_i_*, Akaike weights explaining total variance. Models with Δ*_i_* < 2 are in italics.*

### Apparatus Effect on Mechanical Problem Solving

Friedman ANOVAs revealed an effect of the apparatus on the MPS tasks at 6–7 years of age (χ^2^ = 16.511, *p* < 0.001), 9 years of age (χ^2^ = 10.571, *p* < 0.01), 11–12 years of age (χ^2^ = 10.750, *p* < 0.01) and 13–14 years of age (χ^2^ = 7.429, *p* < 0.05). A significant trend was only seen at 8 years of age (χ^2^ = 5.892, *p* = 0.05), and there were no significant differences between the three apparatus at 10 years of age (χ^2^ = 2.722, *p* = NS).

*Post hoc* intra-group age comparisons showed that the Sloping task was accomplished significantly worse than the two apparatus of the MPS test by 6–7-year-old than 11–12-year-old (*p-values* < 0.05). For the oldest children (13–14 years of age), the scores for the Sloping task were not significantly different from the other apparatus (*p* = 0.07). By contrast, the successful performances with the other two apparatus (Chimney task and Hook task) were equivalent for the six age groups.

## Discussion

The aim of the present study was to explore the typical development of RTU and its relationship with MPS in a sample of school-age children. The novelty of this work lies in the use of a new experimental test to evaluate RTU in everyday-life, associated in the use of three different MPS tasks (Chimney, Hook, and Sloping tasks). In addition, FK was also assessed, with strictly matched items (the same tools were used in the two tasks).

The main finding is a significant developmental progression of scores in children between 6 and 14 years on the RTU test and on the MPS tasks with two apparatus, namely the Hook task and the Sloping task. However, we found only a weak improvement with age for the FK test. Interestingly, MPS appears to be a better predictor of RTU task than FK. Finally, we found no association between the RTU test and FK.

### Underlying Cognitive Processes Involved in the Development of Real Tool Use

We found a significant link between the age of children and the three types of tests proposed in this study: RTU, MPS, and FK. The analysis of relationships between these different forms of tool use abilities revealed in healthy children an interesting and novel involvement of MPS skills in conventional tool use development. More particularly, GLMM analyses revealed that MPS was a good predictor of RTU even when age is included as a random factor. In broad terms, a link between MPS and RTU exists between 6 and 14 years of age. This link supports the hypothesis that the analysis of technical apparatus properties contributes to the development of RTU, independently of age and FK. Along these lines, Gergely and Csibra (2003; and more recently Hernik and Csibra, 2015) developed the notion of a teleogical interpretation system (“teleological stance”) that allows children from age one onward to observe actions and to conjure inferences linking these actions in terms of means and ends as a function of situational constraints. In addition, Hernik and Csibra (2009) point out that an object is always made for something, even if not used. The behavior of even very young children shows this search for an object-function association, providing knowledge on the use and function of objects and allowing the child to learn by observing others.

Regarding the FK test, no developmental effect was found, and the GLMM analysis revealed that FK does not predict RTU. Overall, these results indicate that children possess the majority of FK on these tools from 6 years of age onward. In our study, this knowledge does not contribute to the typical development of the abilities for effective use of these same tools between 6 and 14 years of age. This supports the hypothesis that FK is not necessary for and evolves in parallel to RTU in everyday life. The lack of assoiation between the RTU and FK tests puts the nature of the knowledge examined in question. As a matter of fact, those semantic knowledges allow the selection of the tool that is typically used with a given object. On the other hand, it is unlikely that FK allows for effective and efficient use of the tool in question. More particularly, FK can be used to select a *bottle opener* as the tool usually used to remove the cap from a glass bottle. Nonetheless, this knowledge does not provide the information required to use the tool efficiently (i.e., which part of the tool must be applied to the bottle, what action must be undertaken: lifting, rubbing, turning, etc.). In other words, semantic FK might not be critical for effective use but could facilitate the process to a certain extent (see Hodges et al., 2000; Bozeat et al., 2002). Specific knowledge regarding “use” could be reflected in the “mechanical” properties of the tools (i.e., the way of learning about the tools and the relationship with their abstract physical properties like rigidity, flexibility, etc.).

### Age Effect on Mechanical Problem Solving Depends on the Type of Experimental Apparatus

Our results reveal an effect of age on the performance of the MPS task, particularly for two out of the three apparatus (Sloping and Chimney apparatus) irrespective of the rating method used (i.e., Beck’s rating system or the accuracy-based scoring system created for our study) in agreement with previous study (Beck et al., 2011; Cutting et al., 2011). For the Hook task, our findings show a ceiling effect from 11 years of age onward instead of 16–17 years of age, which could indicate earlier maturity of the underlying processes than in the data of Beck et al. (2011). Several methodological variations could explain these differences (e.g., the time limit, the number of tools). The progression appeared more linear for the Sloping task, with medium to large effect sizes. Performance appeared equivalent between children of 6–7 years of age and 9 years of age (score of 1.1 points out of 3), and an extended improvement of the scores up to 14 years of age was seen (2.3 points, on average, out of 3). By contrast, no significant developmental effect is observed on the Chimney task for the age groups between 6 and 14 years of age. So, the type of experimental apparatus modifies age effects on MPS: mature performance at 6 years of age for the Chimney task, at 11 years of age for the Hook task, or under development at 14 years of age for the Sloping task. Moreover, the details of the accuracy-based scoring system on the Hook task show that from 6 years of age, children get a mean score of 2.1 points out of 3. These results indicate that those who failed to extract the target from the box in the allocated time (47% of the children) were nonetheless able to raise the target off the bottom of the tube. So the analysis of the technical properties of the apparatus already seems to be taking place at the cognitive level. For instance, use of a sufficiently long tool allows contact and action on the target at the bottom of the box (see **Figures 2A,C**). The hypothesis of an early engagement of technical reasoning from age of 6 onward that allows mechanical problems to be solved, nevertheless raises the issue of the cognitive processes explaining the improvement of scores after this age in the MPS tasks.

The two apparatus showing age effects in our sample revealed differential growth curves. In fact, the intra-group analyses revealed a significant effect of the type of apparatus between 6 and 14 years of age. The Sloping task is the only apparatus with considerably less success than the other two up to 11–12 years of age, after which the gap tended to narrow with the older children (13–14 years of age). A partial variation of the scores for solving mechanical problems was hence seen, depending on the different relationships of means-end reciprocity engaged as a function of age: directly reach and move the target for the Chimney task, create a new tool for the Hook task, and carefully explore the apparatus to find the seesaw for the Sloping task. So this partial effect of apparatus that do not share the same technical principles suggests that technical analysis is done in the here and now. This is consistent with the results of Beck et al. (2014), who reported no transfer of knowledge for the Hook task with different means and materials. Another explanation could be that there is an effect of complexity between the different mechanical problems used, since performance in the Sloping task is clearly inferior to that obtained in the other apparatus, whatever the age group. Such an effect of complexity has already been seen among healthy children of pre-school age in the setting of MPS. Thus, Gardiner et al. (2012) revealed a significant difference between the success rates with three types of mechanical problems according to the transparency mechanical relationships between objects present in the apparatus and the degree of tool manipulation required to extract the target from the box. Unlike other apparatus, the Sloping task in our study requires finding the mobile compartment enclosed within the box, so the affordances of this task were partially hidden. The low scores observed in the Sloping task probably reflect the children’s difficulty to readily separate the observable properties of the material so as to discover access to the seesaw located at the back of the box (see **Figure 2D**). So, this particular apparatus could be more subject than the others to the ability to block non-pertinent strategies; namely, to switch and approach the problem from another point of view, and to plan the successive stages required to solve the problem; or, in other words, through use of executive functioning. Given that cognitive processes are also in full development but still relatively immature at the ages covered in our study (Roy, 2015), their contribution could prove to be effective only in older children. This could potentially explain the narrowing of the significant gap between the scores for the Sloping task and the two other mechanical problems with increasing age.

Further research is needed to highlight the nature of the strategies used by children to solve the MPS task. A first step in this direction was through our study with an accuracy-based scoring system, but qualitative analyses based on the *tool selection* (number of relevant and irrelevant tools grasped; see Lesourd et al., 2016) versus *their application* are also required. Such measurements would allow for examination of the capacity of children to recognize the technical means suitable for performing the task (rigid, flexible, sufficiently long, etc.) and not only the adequate form of a preformed tool. The time spent engaged in various actions could also be studied (no handling, tool handling, box handling, tool-box manipulation; see Osiurak et al., 2013) so as to distinguish behaviors exploring the boxes or the tools as separate from genuine attempts at relating to the purpose of the apparatus (extracting the target) through the technical application of the tools. Other studies are also required to explore possible links between MPS and executive functioning in healthy children. While previous works have already raised this issue (Chappell et al., 2013), to our knowledge, no prior study has employed executive tests to address this hypothesis.

## Conclusion

To conclude, this study is original in examining the typical development of RTU and its relationships with MPS. We found an effect of age between 6 and 14 years on the RTU test and on the MPS tasks with only two apparatus. Our findings also provide evidence for the existence of relationships between the real use of tools and MPS, and the joint development of these skills in children. On the other hand, no developmental effect was found for FK test in relation to these tools. Thus, the absence of a relationship with conventional tool use suggests that RTU and FK could evolve in parallel without a strong connection. Furthermore, our results suggest that the underlying processes of technical reasoning (specifying the relationships between various environmental components) are partially operative from the age of 6 onward, even though the outcome of their use varies according to the context in which they are applied. Lastly, while the theoretical framework used in this study is based on work developed using adults, it offers interesting perspectives in regard to understanding the development of abilities of tool use in various contexts in children. The relationships between RTU and MPS remain to be explored in the context of pediatric pathologies, thus potentially allowing the underlying cognitive processes to be dissociated. Studies of children with Developmental Coordination Disorder (DCD) would be particularly relevant, as they experience difficulties with the use of tools in everyday life.

## Author Contributions

CR conceived and designed the study, oversaw the data collected, analyzed and discussed the data, contributed to drafting the manuscript, and reviewed and approved the final version and its submission. Her agreement has been accountable for all aspects of the work. AR participated to the conception of the study, contributed to the analysis of the data, critically reviewed the manuscript, approved the final version to be published, and his agreement has been accountable for all aspects of the work. CJ and FO contributed to the conception of study material, they critically reviewed the manuscript, approved the final version to be published and, their agreement has been accountable for all aspects of the work. OC and DLG critically reviewed the manuscript, approved the final version to be published, and their agreement has been accountable for all aspects of the work.

## Conflict of Interest Statement

The authors declare that the research was conducted in the absence of any commercial or financial relationships that could be construed as a potential conflict of interest.
